# Cost impact of blocking: predictability of ICU patient throughput and cost variance using process modeling

**DOI:** 10.1186/cc9880

**Published:** 2011-03-11

**Authors:** S Nabors, T Bountourelis, A Schaefer, L Luangkesorn, J Kharoufeh, L Maillart, W Yang, G Clermont

**Affiliations:** 1 University of Pittsburgh Medical Center, Pittsburgh, PA, USA; 2 University of Pittsburgh, Pittsburgh, PA, USA

## Introduction

Efficient management of ICU patient turnover can significantly impact patient survival, medical expenses, overall satisfaction, and hospital operating expenses. Movement within a constrained healthcare delivery system is a dynamic and stochastic process that eludes traditional analysis and prediction tools. We hypothesized that simulation-based approaches allow for a better capture of the interaction between reality and policy, and therefore guide efficient ICU management. We developed a simulation process, modeling constrained hospital patient flow in a tertiary care cente and generated a cost-variance analysis derived from differences in that patient flow.

## Methods

This study consists of a retrospective analysis of a comprehensive sample of 3,518 patients admitted to the VA Pittsburgh Health System from 27 April 2010 to 3 November 2010. Patient movement data are extracted to produce a cohort dataset and time-series analysis of patients transitioning in the following units: the medical ICU (nine beds), surgical ICU (11 bed), coronary care unit (18 beds), step-down unit (nine beds), monitored medical (15 beds), monitored surgical (12 beds), nonmonitored medical (44 beds) and surgical (19 beds). Cost data are extracted from the VAPHS annual budget review and cost allocation records for specific patient units and levels of care. Blocking time is the difference between time of assignment and movement to a specified location. Assignment difference is the probability of being assigned to a location other than requested location. Cost variance is the difference between cost allocations based on the standard of clinically indicated LOS and the cost allocations based on real LOS averaged per unit location.

## Results

This model graphically depicts LOS rates, blocking times, assignment difference rates, and cost variance. The worst blocking time is observed for monitored medical beds (44 hours) while the worst assignment difference is observed for surgical monitored beds (0.55). The worst cost variance is recorded in the surgical ICU ($572,000). The total cost variance is $849,000.

## Conclusions

ICU flow is a dynamic process affected by constraints manifesting in large blocking times, assignment differences and significant cost variance. This novel flow management tool could systematically and objectively aid managerial decision-making at both the unit and hospital levels.

